# Enhanced circulating PCSK9 concentration by berberine through SREBP-2 pathway in high fat diet-fed rats

**DOI:** 10.1186/1479-5876-12-103

**Published:** 2014-04-23

**Authors:** Yan-Jun Jia, Rui-Xa Xu, Jing Sun, Yue Tang, Jian-Jun Li

**Affiliations:** 1 Division of Dyslipidemia, State Key Laboratory of Cardiovascular Disease, Fu Wai Hospital, National Center for Cardiovascular Disease, Chinese Academy of Medical Sciences and Peking Union Medical College, Beijing 100037, China

**Keywords:** Berberine, Simvastatin, Proprotein convertase subtilisin/kexin type 9, Low density lipoprotein receptor, High fat diet-fed rats

## Abstract

**Background:**

Berberine (BBR), a natural plant extract, has been shown to improve lipid metabolism. However, its effects on PCSK9, a key factor involving in the lipid metabolism, have not yet been evaluated in vivo. The aim of the present study was to investigate the effect of BBR on PCSK9 expression in high fat diet-fed (HFD) rats.

**Methods:**

Thirty-two male Sprague Dawley (SD) rats were randomized into the four groups (n = 8): normal diet (Control), HFD, HFD + simvastatin (Sim, 2 mg/kg/d) and HDF + BBR (400 mg/kg/d) for 6 weeks. The following parameters were determined: 1) body weight; 2) serum lipid profile; 3) serum PCSK9 measured by enzyme-linked immuno sorbent assay (ELISA) ; 4) hepatic expressions of low-density lipoprotein receptor (LDLR), sterol regulatory element binding protein-2 (SREBP-2) and hepatocyte nuclear factor 1 (HNF1) were examined by real time quantitative polymerase chain reaction (RT-PCR) and western blotting analysis.

**Results:**

Compared with HFD rats, Sim and BBR significantly reduced body weight gain and improved lipid profile (P < 0.05 respectively). In addition, either of drug treatment for 6 weeks could increase serum concentration of PCSK9 in HFD rats (P < 0.05). This enhanced PCSK9 expression was demonstrated to be associated with the up-regulation of hepatic expression of LDLR and SREBP-2 and the down-regulation of hepatic expression of HNF1 (P < 0.05 respectively).

**Conclusions:**

The data provided the first line of the evidence that BBR, similar to the Sim, could increase the expression of PCSK9 levels in HFD rats through SREBP-2 activation, suggesting that impacts of BBR on lipid profile may also be linked to SREBP-2 pathway.

## Introduction

It has been well established that high total cholesterol (TC) and low density lipoprotein cholesterol (LDL-C) are among the most important predictors of future coronary artery disease (CAD) and cardiovascular events [1]. In contrast, reduction in the circulating levels of TC and LDL-C has been demonstrated to significantly reduce the risk for CAD [2]. Therefore, the effective control of dyslipidemia and better understanding of lipid metabolism have been considered as a powerful strategy for the prevention and treatment of CAD [3].

Recent studies of human genetics and genome-wide screens have identified proprotein convertase subtilisin/kexin type 9 (PCSK9) as the third gene associated with autosomal dominant familial hypercholesterolemia, after LDLR and apoB100 [4]. The function of PCSK9, as a secreted serine protease, is degradation of hepatic LDLR, which is directly correlates with its tight association with plasma cholesterol levels and provides a new therapeutic target to combat hypercholesterolemia and CAD [5-8]. At the transcriptional level, PCSK9 has been identified as a target gene of SREBPs [9-11]. The proximal promoter of the PCSK9 gene contains a functional sterol regulatory element (SRE) that responds to changes in intracellular cholesterol levels [12]. In HepG2 cells both SREBP-1 and SREBP-2 transcriptionally activate PCSK9 via this SRE site [9]. In vivo however, it was suggested that the sterol-dependent regulation of PCSK9 is mediated predominantly by SREBP-2 [11].

The most common used lipid-lowering drugs currently is the statins including China. These drugs act as competitive inhibitors of 3-hydroxy-3-methylglutaryl-coenzyme A (HMG CoA) reductase, leading to the reduction of endogenous cholesterol synthesis, depletion of intracellular cholesterol levels and a subsequent up-regulation of the low-density lipoprotein receptors (LDLRs) through the sterol regulatory element binding protein (SREBP) pathway [3,4]. In addition, an increased hepatic LDLR expression can result in the improvement in clearance of plasma LDL-C [5-11].

Recently, statins have also been shown to enhance the expression of PCSK9 gene through the SREBP pathway [12]. Several studies have reported that statin treatment not only decreases LDL-C and improves cardiovascular adverse events but also causes an increase in the concentration of serum PCSK9 [13-17]. Moreover, it was postulated that statins increased the activity/nuclear translocation of SREBP-2, resulting in the increased expression and secretion of the PCSK9 protein. Meanwhile, several other studies have also indicated that another commonly prescribed lipid-lowering agent, fenofibrate, is also reported to increase the circulating levels of PCSK9 [18,19].

Berberine (BBR) is an isoquinoline plant alkaloid and has long been used in Chinese medicine [20-23]. BBR has commonly been used as a traditional herbal medicine to treat bacterial infection and many other illnesses without toxic effects reported to date in a body of preclinical and clinical studies [20,21]. Interestingly, BBR has also been demonstrated to lower fasting triglyceride (TG) levels in a clinical trial and reduce body weight as well as improve dyslipidemia in high fat diet-fed (HFD) rats. Hence, the understanding the cardiovascular protective effects of BBR appears to be mounting [22,23].

The aim of the present study, therefore, was to evaluate the effects of BBR on plasma PCSK9 concentration as well as its potential mechanism using HFD rat model.

## Methods

### Drugs and regents

BBR Chloride was purchased from haoran biological technology company (shanghai city, China). Simvastatin (Sim) was kindly provided by Merck Research Laboratories (Hangzhou city, China). BBR or SS were dissolved in 0.5% sodium chloride before use.

### Animal studies

After weaning, 3-week male Sprague–Dawley (SD) rats (average body weight, 50 ± 1 g) were purchased from animal center of Beijing University Health Science Center. All animals were placed in cage (four rats per cage) in an atmosphere of 55 ± 10% relative humidity at 22 ± 2°C with a 12 h light/dark cycle. Rats were given free access to food and water. The study protocol was approved by the ethics committee on Animal Center of Beijing University Health Science.

After adapting to the environment for a week, animals were randomly assigned into the four groups (n = 8 for each group): Control (Con) group, HFD group, HFD + Sim group, HFD + BBR group. The indicating diets were purchased from Beijing keao Feed Company. Rat in Con group was fed with a standard chow diet (3.21 kcal/g, 13% calories from fat, 25% calories from protein, 62% calories from carbohydrate) for 6 weeks. The HFD group was fed with a HFD (4.39 kcal/g, 47% calories from fat, 20% calories from protein, 33% calories from carbohydrate) for 6 weeks. The HFD + BBR group was fed with a HFD plus BBR (400 mg/kg/d, oral input) for 6 weeks. The HFD + Sim group was fed with a HFD plus Sim (2 mg/kg/d, oral input) for 6 weeks. During experiments, rats had free access to water and food and food consumption was measured every day for the purpose of excluding the changes of body weight or lipid profile due to the variation of caloric intake. At the end of the experiment, all animals were anesthetized with ether and the blood samples were collected in EDTA-coated tubes and centrifuged. The plasma and livers were rapidly collected and stored at -80°C until analysis.

### Body weight measurement and biochemical analysis

Animal’s body weight was measured in every week from week 0 (before treatment) to week 6 (the end of the study).

Fasting blood samples were collected and plasma levels of TC, TG, high density lipoprotein cholesterol (HDL-C) were determined on an automatic biochemistry analyzer (Hitachi 7150, Tokyo, Japan). The LDL-C was estimated according to the following formula: LDL-C = TC-HDL-C-TG/5.

### Assay of plasma PCSK9 concentration

PCSK9 concentrations were measured using a high-sensitivity, quantitative sandwich enzyme immunoassay (Quantikine ELISA, R&D systems Europe Ltd, Sweden) according to our previous publications [14,15]. The lower limit of detection was 0.096 ng/ml. According to the manufacturer, the inter-assay coefficient of variation is less than 5%.

### Detection of hepatic LDLR, SREBP-2 and hepatocyte nuclear factor-1 (HNF-1) mRNA expression

Key enzymes of lipid metabolism, which were analyzed by real time quantitative polymerase chain reaction (RT-PCR), were selected as candidate genes for the assessment of their mRNA expression levels in the liver of rats studied (Table 1). Briefly, total RNA in liver tissue was isolated from hepatic tissue using the Trizol reagent Kit (Invitrogen, USA) following the manufacture's instructions. And then, it was measured by spectrophotometry at an absorbance of 260 nm, and designated the purity valid if the ratio of A260/A280 was in the range from 1.8 to 2. The integrity of the RNA was checked by denaturing agarose gel electrophoresis and ethidium bromide staining. 3 μg of the total RNA was reversed transcribed by reverse Aid First Strand cDNA synthesis kit (Fermentas, CA, USA). The abundances of key genes (LDLR, SREBP-2 and HNF-1) and glyceraldehyde-3- phosphate dehydrogenase (GAPDH) mRNA were analyzed by RT-PCR using the 7500HT RT-PCR system (Applied Biosystems, Foster, CA, USA). RT-PCR was performed using the SYBR Premix ExTaq (TaKaRa Bio Inc. Japan) according to the manufacturer's instructions.

**Table 1:** The Sequences of primers for Real-Time PCR used in the study

**Gene description**	**Primer**	**Sequences (5' → 3')**
**LDLR**	**F**	GATTGGCTATGAGTGCCTATGTC
	**R**	GTGAAGAGCAGAAACCCTATGG
**SREBP-2**	**F**	AGCATACCGCAAGGTGTTCC
	**R**	CCAGGTGTCTACTTCTCCGTGT
**HNF1**	**F**	ATGACACGGATGACGATGGG
	**R**	ATGGGTCCTCCTGAAGAAGTGA
**GAPDH**	**F**	ACAGCAACAGGGTGGTGGAC
	**R**	TTTGAGGGTGCAGCGAACTT

PCR = polymerase chain reaction; F = forward; R = reverse; LDLR = low density lipoprotein receptor; PCSK9 = proprotein convertase subtilisin/kexin type 9; SREBP-2 = sterol regulatory element binding protein-2; HNF1 = Hepatocyte Nuclear Factor 1; GAPDH = reduced glyceraldehyde-phosphate dehydrogenase.

Standard curves for each primer pair were generated by serial dilutions of cDNA from a reference sample and used for regression analyses. All PCR assays were performed in triplicate. The variance of the triplicate measurements was <1%. Results were analyzed using the standard curve method by the sequence detection systems (SDS) software. The data was expressed as the relative levels of mRNA after normalized with GAPDH.

### Western blot analysis of LDLR, SREBP-2 protein expression

Western blot analysis was performed to examine the protein expression in the liver tissue of rats studied. Briefly, the liver sample (0.2 g) were homogenized in 3 ml of lysing buffer (25 mM MES, pH 6.5, 0.15 M NaCl, 1% v/v Triton X-100, 60 mM octylglucoside, and 1X protein inhibitors) using a Dounce homogenizer. After incubation on ice for 20 min, homogenates were centrifuged at 12,000 g for 15 min at 4°C. Supernatants were collected and protein concentration was measured using a BCA protein detection kit. We separated the proteins on 10% SDS polyacrylamide gels under reducing conditions and transferred them, eletrophoretically, onto PVDF membranes (Bio-Rad, USA). Subsequently, membranes were blocked in 10% bovine serum albumin in Tris buffered saline with 0.1% Tween 20 for 30 minutes. Membranes were incubated with primary antibody (anti-LDLR antibody, anti-SREBP-2 antibody and β-Actin antibody) at 4°C for overnight. After three washings with Tris buffered saline containing 0.1% Tween 20 for 15 minutes, membranes were incubated with second antibody at a 1:1000 dilution for 30 minutes. After washing, the membranes were developed with ECL western blotting reagents according to the manufacturer’s instructions. Films were scanned and quantified by using Image-Quant software (Molecular Dynamics).

### Statistical analysis

Results are expressed as the means ± standard deviation (SD). Statistical analysis was performed using SPSS 19.0 statistical packages (Chicago, Illinois, USA). The significance of differences was evaluated by a one-way analysis of variance (ANOVA) for unpaired data. The significance level was chosen as P < 0.05.

## Results

### Changes of body weight and plasma lipid levels induced by Sim and BBR

Rats fed with HFD tended to developing obesity, shown by increased body weight compared with controls, while either Sim or BBR could decrease the body weight of rats fed with HFD (P < 0.05 respectively). The body weight loss was very significant following a 6-week treatment of Sim or BBR (Table 2). Besides, as shown in Table 2, the 6-week HFD regimen significantly increased serum TG, TC, LDL-C and reduced serum HDL-C levels in HFD rats compared with control rats (P < 0.05 and P < 0.01 respectively). However, a 6-week treatment of BBR (400 mg/kg/d) and Sim (2 mg/kg/d) significantly decreased the serum levels of TG (P < 0.05 and P < 0.01 respectively). Meanwhile, Sim significantly decreased serum TC and LDL-C (P < 0.05). Both Sim and BBR could increase the serum levels of HDL-C, while BBR was more prominent in decreasing serum TG concentration and increasing HDL-C concentration compared with Sim group (P < 0.01 respectively).

**Table 2:** Changes of body weight and plasma lipid profile

	**Con**	**HFD**	**HFD + Sim**	**HFD + BBR**
	(n = 8)	(n = 8)	(n = 8)	(n = 8)
Body weight (g)	325 ± 5.8	405 ± 4.4^*^	345 ± 9.4^#^	360 ± 8.7^#^
TC (mmol/L)	2.20 ± 0.07	2.33 ± 0.12	2.10 ± 0.12^#^	2.28 ± 0.12
TG (mmol/L)	0.70 ± 0.008	1.80 ± 0.003^*^	1.20 ± 0.015^#^	0.80 ± 0.019^##^
LDL-C (mmol/L)	0.46 ± 0.02	0.93 ± 0.03^*^	0.56 ± 0.04^#^	0.52 ± 0.04^#^
HDL-C (mmol/L)	1.49 ± 0.04	1.10 ± 0.03	1.32 ± 0.10	1.60 ± 0.02^##^

Con = control; HFD = high fat diet; HFD + Sim = high fat diet + simvastatin; HFD + BBR = high fat diet + berberine; TC = total cholesterol; TG = triglyceride; LDL-C = low density lipoprotein cholesterol; HDL-C = high density lipoprotein cholesterol. *<0.05 vs Con; ^#^<0.05 vs HFD; ^##^<0.01 vs HFD.

### Elevation of plasma PCSK9 levels

**Figure 1:**
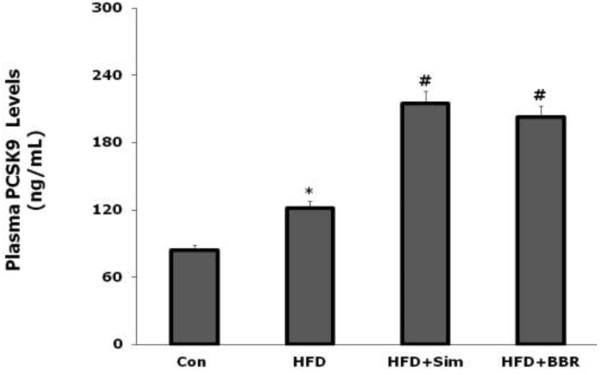
**Effects of simvastatin and berberine on plasma PCSK9 concentration in rats at 6 weeks assessed by ELISA.** Values are mean ± SD, n = 8. ^*^P < 0.05, HFD vs Con; ^#^P < 0.05, HFD + BBR or HFD + Sim vs HFD. Con = control; HFD = high fat diet; HFD + BBR = HFD + berberine; HFD + Sim = HFD + simvastatin.

As shown in Figure 1, plasma PCSK9 levels were significantly higher in rats fed with HFD compared to that in rats fed with normal diet (P < 0.05), suggesting that the HFD could up-regulate the expression of PCSK9. More interesting, rats treated by BBR showed a higher plasma PCSK9 levels, which was similar to that treated by Sim compared with rat fed with HFD alone (P < 0.05).

### mRNA expression of hepatic LDLR, SREBP-2 and HNF1

**Figure 2:**
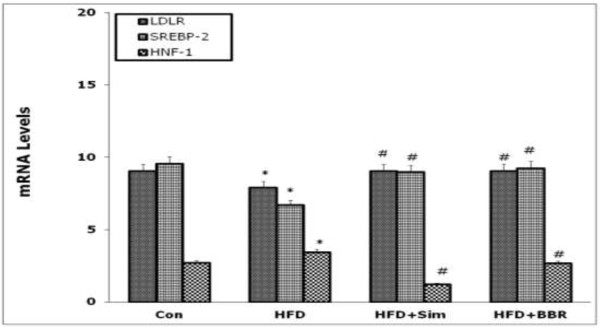
**Effects of simvastatin and BBR on LDLR, SREBP-2 and HNF1 mRNA expression in the liver tissues at 6 weeks assessed by RT-PCR.** Data were normalized to the GAPDH mRNA levels and then compared to control group or HFD group measurements. Values are mean ± SD, n = 3. *P < 0.05, HFD vs Con; #P < 0.05, HFD + Sim or HFD + BBR vs HFD. Con = control; HFD = high fat diet; HFD + BBR = HFD + berberine; HFD + Sim = HFD + simvastatin; LDLR = low density lipoprotein receptor; SREBP-2 = sterol regulatory element binding protein-2; HNF1 = Hepatocyte nuclear factor 1; RT-PCR = Real time quantitative reverse polymerase chain reaction.

As presented in Figure 2, Sim and BBR treatment for 6 weeks significantly increased the levels of hepatic LDLR and SREBP-2 mRNA in rats fed with HFD compared with that in rat fed with HFD alone (P < 0.05). Meanwhile, Sim and BBR treatment remarked decreased the levels of hepatic HNF1 mRNA in rats fed with HFD compared with that in rat fed with HFD alone (P < 0.05).

### Protein expression of hepatic LDLR, SREBP-2

**Figure 3:**
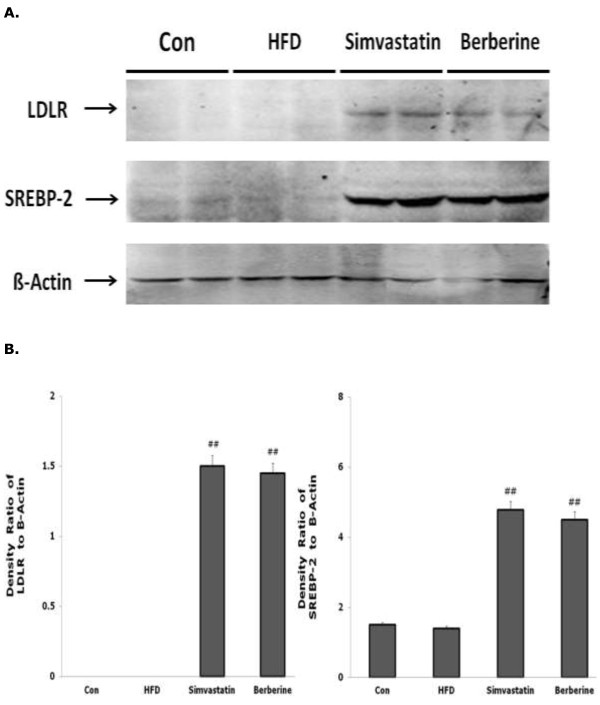
**Effects of simvastatin and BBR on LDLR and SREBP-2 protein expression of the liver tissues at 6 weeks assessed by western blotting analysis. A**. A representative western blot analysis is shown; **B**. Western blots were quantitated as described in methods. Con = control; HFD = high fat diet; Simvastatin = HFD + simvastatin; Berberine = HFD + berberine; LDLR = low density lipoprotein receptor; SREBP-2 = sterol regulatory element binding protein-2.

The pattern of protein expression of hepatic LDLR, SREBP-2 was very similar to the mRNA expression in our present study. As summarized in Figure 3, Sim and BBR treatment for 6 weeks also significantly increased the expression of hepatic LDLR and SREBP-2 protein in rats fed with HFD compared with that in rat fed with HFD alone (P < 0.01).

## Discussion

The main findings of the present study are: (1) Sim and BBR decreased body weight and improved plasma lipid profile in HFD rats; (2) Sim and BBR increased circulating PCSK9 levels in HFD rats; (3) Sim and BBR increased mRNA levels of LDLR and SREBP-2 but decreased mRNA levels of HNF1 in the liver of HFD rats. (4) Changes of mRNA levels of LDLR and SREBP-2 in the liver of HFD rats were in consistent with that at protein levels, suggesting that enhanced circulating PCSK9 concentration by berberine was associated with SREBP-2 pathway in high fat diet-fed rats. Thus, the present study provided novel information regarding the impact of BBR on HFD-induced obesity and lipid disorders in vivo.

Obesity and overweight have been shown to be associated with cardiovascular diseases (CVD) such as arteriosclerosis, stroke and myocardial infarction, which were closely associated with lipoprotein metabolism [22]. It has been well recognized that the body weight gain can elevate TC, TG and LDL-C levels and reduce HDL-C level. Conversely, the weight loss is associated with lower levels of TG and LDL-C and higher levels of HDL-C, which can confer to cardiovascular benefit. Thus, the control of body weight is very important to affect cardiovascular health [23]. In the present study, the data indicated that BBR and Sim could significantly reduce the body weight and improve the lipid profile in rats fed with HFD compared with that in rats fed with HFD alone (P < 0.05).

During the past few years, it has become clear that the major classes of usually prescribed lipid-lowering drugs such as statins elevated circulating PCSK9 levels [14,15]. Moreover, previous studies suggested that PCSK9 and LDLR both contain an SRE motif in their proximal promoters and thus are coordinately up-regulated by statins through activation of SREBP [13-16,24,25]. Alborn et al. found that there was a strong correlation of LDL-C and PSCK9 levels in humans [26]. Several studies have demonstrated the induction of PCSK9 by statins in cultured cells and in animal models [26]. In human studies, Careskey and colleagues firstly in 2008 reported that atorvastatin could increase circulating PCSK9 levels by the activation or nuclear translocation of SREBP-2, a transcription factor that activates both the LDLR and PCSK9 genes [18]. Subsequently, the impact of lipid-lowering drugs on plasma PCSK9 concentration has intensively been investigated [14-17,20-33]. This effect of Sim on plasma PCSK9 was also identified in our present animal study. Thus, findings from in vitro and in vivo studies raised an important question whether the PCSK9 gene transcription could be separately regulated from LDLR.

It has been demonstrated that BBR improved lipid profile by post-transcriptional mechanism, which involves the activation of the extracellular signal-regulated kinase (ERK) signaling pathway [30]. In recent years, the studies have also indicated that BBR can down-regulate the transcription of PCSK9 while up-regulate LDLR mRNA level post-transcriptionally in HepG2 cells [20,30]. This investigation led us to uncover a novel transcriptionally regulatory role that modulated the expression of PCSK9 in vivo. In the present study, data showed, for the first time, that the mRNA levels of PCSK9, LDLR and SREBP-2 of the livers in HFD rats were significantly up-regulated while that of HNF1 was down-regulated by BBR, which was also demonstrated at protein levels assessed by western blotting analysis.

PCSK9, a serine protease expresses in the liver and intestine, which moves to the cell surface after auto-cleavage in endoplasmic reticulum (ER) and is secreted into the plasma [31]. The role of PCSK9 as post-transcriptional regulator of the amount of LDLRs in the liver is supported by the finding that gain of function mutations of PCSK9 in humans could cause hyperocholesterolemia, resulting in a increase of cardiovascular risk [31]. It has been indicated that the promoter region of PCSK9 gene contains the HNF1 and SRE sites, which respectively bind the trans-activation proteins HNF1 and SREBP2. HNF1 is essential for PCSK9 basal transcription and for SREBP2- induced maximal gene expression in response to intracellular cholesterol depletion [32].

In the present study, in order to elucidating the effects of BBR on expression of PCSK9, we provide new evidence to support the notion that lipid drugs increase of PCSK9 and LDLR expression in the process of lipid-lowering treatment. This led us to speculate how BBR affects expression of PCSK9 gene and what interactions of both SREBP and HNF1 factors are involved. By RT-PCR analysis we first showed that the expression of PCSK9 was higher in BBR- and Sim-treated rats compared with that in rat fed HFD alone (P < 0.05). Meanwhile, the enhanced expression of LDLR and SREBP-2 were also found in BBR-treated and Sim-treated rats compared that with HFD rat alone (P < 0.05). In contrast, the expression of HNF1 was lower in BBR-treated and Sim-treated rats compared with that in HFD rats (P < 0.05). The data imply an effect occurred in BBR-treated rats by down-regulation of HNF1 transcription factor at modest levels. This observed would be beneficial with regard to LDLR expression because SREBP2 is absolutely required for LDLR transcription, and a strong improved expression of SREBP2 would eventually increased LDLR expression. The facts that BBR treatment increases LDLR protein level in HFD rats suggested that the balanced effects of BBR are in favor of LDLR expression and stability. In additional, BBR-treatment increased the expression of PCSK9 similar to Sim in HFD rats. Interestingly, for the first time, we showed that BBR also up-regulated PCSK9, LDLR and SREBP-2 in HFD rats. Genevieve’s study found that the regulation of hepatic LDLR expression by statins is multifactorial including transcriptional activation through SREBP-2 and post-transcriptional modulational modulations by PCSK9 [16,26]. It is possible, statin increased hepatic LDLRs that would act to bind the PCSK9 in the circulation. Further increased expression of PCSK9 protein might exceed LDLR binding, resulting in increased circulating levels of PCSK9 protein and only a modest additional decrease in LDL-C [17]. LDLR and SREBP-2 mRNA up-regulation translates into increased circulating PCSK9 protein, adding a PCSK9 inhibitor to statin therapy presents the possibility of further lowering LDLC to recommended concentrations in patients unable to attain desired LDLC on statin therapy alone [34]. Thus, our study confirmed previous studies and provided novel additional important information regarding the lipid-lowering drugs on plasma PCSK9. Given that statins and BBR improved lipid metabolism levels in HFD and increase PCSK9 at the same time, it is reasonable to infer that a combination of BBR and an inhibitor of PCSK9, such as monoclonal antibody targeting PCSK9, would bring greater lipid-lowering effects in the future.

## Conclusion

The present study demonstrated that BBR could reduce the body weight and improve the blood lipid profile of rat fed with HFD through up-regulating the expression of SREBP-2, suggesting that impacts of BBR on lipid profile might also be linked to SREBP-2 pathway. Further studies are required to determine the clinical significance involved in the up-regulation of PCSK9 by BBR.

## Competing interests

The authors declare that they have no competing interests.

## Authors' contributions

YJJ carried out the animal study, analyzed the data, and drafted the manuscript. RXX and JS participated in the measurement and analysis of plasma PCSK9 and gene expressions. JJL and YT conceived and designed the study, supervised all the experiments, interpreted the data, and edited the manuscript. All authors read and approved the final manuscript.
